# Surgical Clips Migration up to Renal Collecting System from Ileal Conduit Postcystectomy

**DOI:** 10.1089/cren.2016.0121

**Published:** 2016-12-01

**Authors:** Hani Albadawi, Tarik Emre Sener, Saeed Bin Hamri

**Affiliations:** ^1^ Department of Urology, King Abdulaziz National Guard Hospital, Riyadh, Saudi Arabia.; ^2^ Department of Urology, Marmara University, Istanbul, Turkey.; ^3^ PETRA-Urogroup, Paris, France.

**Keywords:** flexible ureterorenoscopy, laser, cystectomy

## Abstract

This is a 49-year-old female known to have had cystectomy and ileal conduit 4 years ago presented to our hospital complaining of left flank pain with recurrent urinary tract infection. Radiologic investigations showed left lower pole renal stone. She underwent left laser flexible ureterorenoscopy. Renal collection system was fully explored that showed stone occupying the lower calix, laser disintegration of the stone revealed what we assumed are surgical clips.

## Introduction

Urinary diversion has well-known morbidity and mortality rates. Major complications have been reported up to 56% in ileal conduits and up to 57% in continent catheterizable pouches.^1^ Bacteriuria was found in 50%–90% of urinary diversions that lead to increase the risk of urinary stone formation.^1^ Managing these stones represents a real challenge. Huge advances in endourologic miniaturizations increased the tendency of stone treatment. Retrograde approach in urinary diversions may be technically feasible using ureteral access sheath.^2^ The incidence of renal stones secondary to surgical clips from cystectomy and ileal conduit has not been reported; to our knowledge this is the first reported case of such identity.

## Case Report

**FIG. 1.**
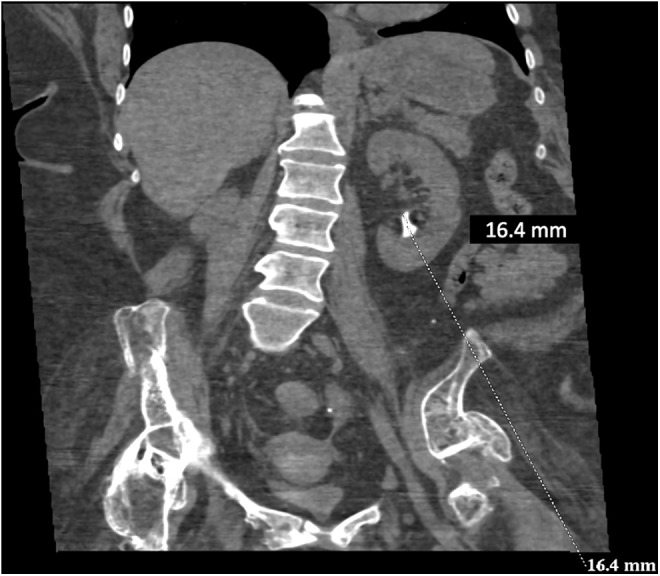
CT scan showing the left renal stone.

**FIG. 2.**
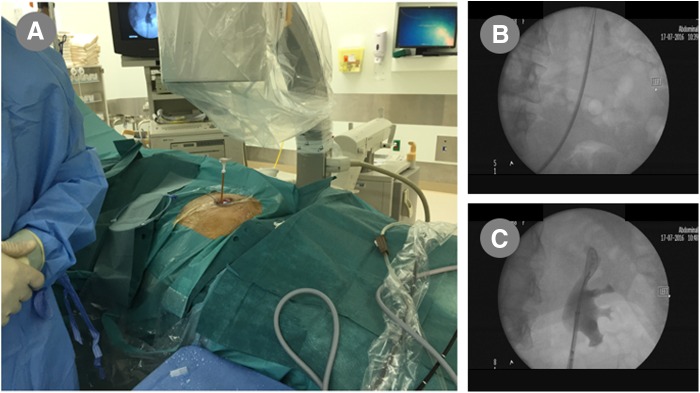
**(A)** Ureteral access sheath through ileal conduit up to the upper ureter, **(B)** ureteral access sheath (fluoroscopy) and radiopaque stone, **(C)** renal collecting system full exploration.

**FIG. 3.**
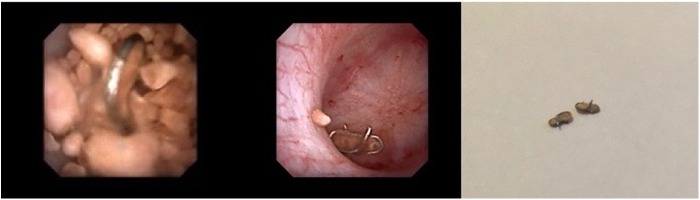
Surgical clips were found postlaser disintegration of left renal stone.

This is a 49-year-old female known to have cystectomy and ileal conduit in 2012. She presented to our clinic complaining of left recurrent flank pain with recurrent urinary infections. Physical examination revealed a good ileal pouch. Renal function test was within normal. Abdomen CT scan showed left lower pole renal stone with a burden of 16.4 mm (Fig. 1). The patient underwent left laser flexible ureterorenoscopy (L-FURS) through her ileal conduit (Fig. 2). Stone laser fragmentation was completely achieved, surprisingly the stone was formed over what we assume were surgical clips from the previous surgery (Fig. 3).

## Discussion

Urinary stone is a real dilemma in patients with urinary diversions. Shock wave lithotripsy, retrograde intrarenal surgery, and percutaneous nephrolithotomy were commonly used. With the advances in endourology, retrograde access is now commonly used to treat such pathology.^2^ Regular follow-up is mandatory to prevent recurrence; moreover, metabolic changes have to be observed closely. Nevertheless, there are some reported cases of migration of surgical clips into the upper urinary tract as nidus for stone formation.^3–5^ Our patient's renal stone formation over surgical clips postcystectomy with ileal conduit is the first in the world.

## Conclusion

Retrograde L-FURS procedure in patients presenting with renal stone as late complication of urinary diversion is safe with low morbidity rates. Metal clips may migrate postoperatively and cause complications such stone formation as well as infection. Therefore, they should be avoided and applied selectively; moreover, urologists should think of this as a cause of renal stone posturinary diversions.

## Abbreviations Used

CT: computed tomography
L-FURS: laser flexible ureterorenoscopy
